# Characterizing the role of SLC3A2 in the molecular landscape and immune microenvironment across human tumors

**DOI:** 10.3389/fmolb.2022.961410

**Published:** 2022-08-05

**Authors:** Jiajun He, Dong Liu, Mei Liu, Rong Tang, Dongqing Zhang

**Affiliations:** ^1^ Minhang Hospital, Fudan University, Shanghai, China; ^2^ The First Affiliated Hospital of Soochow University, Suzhou, China; ^3^ Shanghai Medical College, Fudan University, Shanghai, China

**Keywords:** SLC3A2, cancer, ferroptosis, immune microenviroment, pan-cancer

## Abstract

**Background:** Inducing ferroptosis in human tumors has become a potential strategy to improve the prognosis of patients, even in those with chemotherapeutic resistance. The xCT complex is a major target for ferroptosis induction, constituted by SLC7A11 and SLC3A2. The role of SLC7A11 in cancer has been widely studied in recent years. However, related research studies for its partner SLC3A2 are still rare.

**Methods:** Bulk transcriptome, single-cell sequencing, and immunohistochemical staining were analyzed to explore the expression distribution of SLC3A2. Clinical outcomes were referred to uncover the relationship between SLC3A2 expression and patients’ prognosis. Immune cell infiltration was estimated by multiple deconvolution algorithms. The effect of SLC3A2 on the proliferation and drug resistance of cancer cell lines was evaluated by DEPMAP.

**Results:** Upregulated SLC3A2 may have an adverse effect on the survival of multiple cancers such as lower-grade glioma and acute myeloid leukemia. SLC3A2 expression is indispensable for multiple cell lines’ proliferation, especially for ESO51 (a cell line for esophageal cancer). In addition, SLC3A2 expression level was related to the remodeling of the immune microenvironment in cancers and some immune checkpoints such as PD-1 and PD-L1, which were potential therapeutic targets in many distinct cancers.

**Conclusion:** Our study systematically elucidated the role of SLC3A2 in the survival of cancer patients and the potential immunotherapeutic response. Few molecular mechanisms by which SLC3A2 regulates anti-tumor immunity have been clarified in the present study, which is the main limitation. Future research into the biological mechanism could further help with targeted treatment for cancer patients.

## Introduction

Cancer is a leading cause of death worldwide and afflicts millions of people (Bray et al., 2018). Multimodal therapies, such as chemotherapy and immunotherapy, have been applied to improve overall survival to some extent, which is the final goal of any cancer-directed treatment (Chen et al., 2017; Riley et al., 2019). Despite the significant advances in cancer treatments, the management of patients faced with chemotherapeutic resistance remains dramatically tough. Ferroptosis is a novel cell death mechanism characterized by excessively accumulated lipid peroxidation, that has been reported to be effective in curing tumors with chemotherapeutic resistance (Hassannia et al., 2019). Targeting ferroptosis is expected to benefit numerous patients with cancer.

Cystine/glutamate exchanger (xCT) is an important molecule mediating ferroptosis resistance in cancer cells, which is comprised of SLC7A11 and SLC3A2. While the role of SLC7A11 in cancer biology has been studied widely in recent years, few studies have focused on the effect of SLC3A2 during tumorigenesis and its association with patients’ prognosis. Sun et al. have reported that overexpression of SLC3A2 on cell membrane contributed to the accelerated proliferation of oral squamous cancer cells, while knockdown of SLC3A2 could counteract tumor cell invasion and migration (Liang and Sun, 2021). Mechanistically, SLC3A2 promoted the aggressive phenotype by facilitating the expression of several mucin genes in gastric cancer (Wang et al., 2017). The intra-cellular level of SLC3A2 could be regulated by post-transcription modification like m6A-dependent degradation (Ma et al., 2021). Overall, these *in vitro* studies preliminarily suggest that SLC3A2 may be significant in tumor initiation and progression. Nonetheless, a comprehensive analysis of the role of SLC3A2 in cancers based on human genetic resources was still lacking, which may hinder further in-depth translational research.

Here, we conducted a pan-cancer level study to systematically analyze the role of SLC3A2 in patients with cancer. First, the association between SLC3A2 expression and patients’ prognosis was analyzed. Then, the disturbance of molecules and microenvironment associated with the SLC3A2 level was revealed. Moreover, SLC3A2 expression and the intra-tumoral mutation landscape were also investigated in the present study.

## Materials and methods

### The source of genome sequencing, bulk and single-cell transcriptome, clinical information, and gene effect for cell-line proliferation

The bulk transcriptome data of 33 cancers, including adrenocortical carcinoma (ACC), bladder urothelial carcinoma (BLCA), breast invasive carcinoma (BRCA), cholangiocarcinoma (CHOL), colon adenocarcinoma (COAD), cervical squamous cell carcinoma and endocervical adenocarcinoma (CSEA), lymphoid neoplasm diffuse large B cell lymphoma (DLBC), esophageal carcinoma (ESCA), glioblastoma multiforme (GBM), head and neck squamous cell carcinoma (HNSC), kidney chromophore (KICH), kidney renal clear cell carcinoma (KIRC), kidney renal papillary cell carcinoma (KIRP), acute myeloid leukemia (LAML), brain lower grade glioma (LGG), liver hepatocellular carcinoma (LIHC), lung squamous cell carcinoma (LUSC), lung adenocarcinoma (LUAD), mesothelioma (MESO), ovarian serous cystadenocarcinoma (OV), pheochromocytoma and paraganglioma (PCPG), pancreatic adenocarcinoma (PAAD), prostate adenocarcinoma (PRAD), rectum adenocarcinoma (READ), sarcoma (SARC), skin cutaneous melanoma (SKCM), testicular germ cell tumors (TGCT), thyroid carcinoma (THCA), stomach adenocarcinoma (STAD), thymoma (THYM), and uterine corpus endometrial carcinoma (UCEC), uterine carcinosarcoma (UCS), uveal melanoma (UVM), were all downloaded from The Cancer Genome Atlas (TCGA) database. Due to the lack of normal samples in TCGA, we also included the transcriptome data from the Genotype-Tissue Expression (GTEx) (https://www.gtexportal.org/home/index.html), which is a comprehensive public resource to study tissue-specific gene expression. In addition, the expression level of SLC3A2 is also evaluated in distinct cancer cell-lines through the Cancer Cell Line Encyclopedia (CCLE) (https://portals.broadinstitute.org/ccle/data). We selected fragments per kilobase million (FPKM) as the data format for the following calculation. The patients’ clinical features containing overall survival (OS), disease-specific survival (DSS), disease-free interval (DFI), and progression-free interval (PFI) were also downloaded from the TCGA database and matched to transcriptome data.

### Differential expression analysis

A unified standardized pan-cancer dataset integrating the TCGA and GTEX databases were downloaded from UCSC (https://xenabrowser.net/). Wilcoxon Rank Sum and Signed Rank Tests were used to evaluate the statistical significance of the differential expression of SLC3A2 between tumor and normal tissue. Given all transcriptome data experienced a log2 (x + 1) transformation, *p* values less than 0.05 of the Wilcoxon test was seen to be significant for differentially expressed SLC3A2.

### Immunohistochemical staining

The IHC data were collected from the human protein atlas (https://www.proteinatlas.org/). The antibody CAB010455 was chosen for further analysis.

### Prognostic relevance

A univariate Cox regression analysis was used to identify the relationship between SLC3A2 expression level and OS, DSS, DFI, and PFI across 33 cancers. Furthermore, hazard rate (HR) was applied to evaluate the magnitude of association with the “survival” package in R software (version: 3.1–8). Kaplan-Meier survival curve was depicted to visualize the associations with statistical significance. The R package “maxstat” (version:0.7-25) was used to determine the optimal cut-off value for the distinguishment of high- and low-risk groups.

### Immune infiltration estimation

The R package “Estimate” (version: 1.013) was used to estimate the proportion of immune and stromal cells in malignant tumor tissues from transcriptome data according to “immune score”. The estimate score equals to the sum of immune and stromal scores. Pearson correlation coefficient (r) was applied to access the strength of the association between SLC3A2 expression level and immune/stromal/estimate scores. The expression level of 150 immunostimulatory genes and 60 immune check-point genes were extracted from the transcriptome data of each cancer. The co-expression association was also calculated using the Pearson correlation coefficient and visualized as a heatmap.

The infiltration of six common immune cells, including CD8^+^ T cells, CD4^+^ T cells, B cells, macrophages, neutrophils, and dendritic cells, was evaluated using the Tumor Immune Estimation Resource (TIMER) database. Other algorithms including CIBERSORT, QUANTISEQ, XCELL, EPIC, MCPCOUNTER, and IPS were also performed according to respective guidance (Newman et al., 2015; Becht et al., 2016; Aran et al., 2017; Charoentong et al., 2017; Li et al., 2017; Racle et al., 2017).

To investigate the single-cell transcriptomic expression of SLC3A2, we applied the TISCH method to analyze the expression pattern of SLC3A2 in different types of cells in the tumor microenvironment (http://tisch.comp-genomics.org/).

To further study the relationship between SLC3A2 expression and T-cell dysfunction and immunotherapeutic response, we turned to the TIDE algorithm and performed relevant analysis (http://tide.dfci.harvard.edu/).

### Genome analysis

Tumor mutant burden (TMB) is defined as the total number of somatic gene coding errors, base substitutions, and gene insertions or deletions detected per million bases. We calculated the TMB of each cancer sample based on the exome sequencing data from the TCGA database (VarScan2) using the maftools package. MSI, which stands for microsatellite instability, refers to the occurrence of new microsatellite alleles due to any change in the length of a microsatellite or due to the insertion or deletion of duplicate units in a tumor as compared to normal tissue. We referred to previous studies for summarized MSI data across distinct cancers. Neoantigens are encoded by mutated genes of tumor cells, which are mainly abnormal proteins produced by gene point mutation, deletion mutation, and gene fusion that are different from those expressed by normal cells. The function “inferHeterogeneity” was used to calculate mutant-allele tumor heterogeneity. The HRD, LOH, and ploidy for each sample were downloaded from a previous study (Thorsson et al., 2018). We analyzed the level of neoantigen through TCGA exome sequencing data.

### Methylation regulators and SLC3A2 expression

Transcriptome data of 44 methylation regulators were extracted from the RNA sequencing data of each cancer (m1A (10), m5C (13), m6A (21). The correlation between these genes and SLC3A2 was evaluated by Pearson correlation.

### Cell culture and viability detection

HCT116 and Panc-01 cell lines were cultured in DMEM or RPMI‐1640 (Gibco, United States) supplemented with 10% fetal bovine serum (Gibco, United States) and 1% antibiotics (penicillin 10,000 U/ml, streptomycin 100 mg/ml) (Solarbio, China). The cells were maintained at 37°C in a humidified atmosphere of 5% CO_2_.

Cells were seeded in 96-well plates in a 100 μL medium and treated with erastin (0-10 uM) (MCE, China). 10 μl Cell counting kit-8 solution (Bimake, China) was added to each well and incubated at 37°C for 2 h. The absorbance at 490 or 450 nm was measured on a spectrophotometer.

### RNA interfering and qRT-PCR

The sequence of siRNA used to interfere SLC3A2 was 5′-AGAUG AAGAUAGUCAAGAA-3 (forward), and 5′-UUCUUGACUAUCUUCAUCU-3′(reverse). Lipofectamine 3000 (Thermo Fisher Scientific) was used for siRNA transfection according to the manufacturer’s guidelines. Briefly, when the cells reached around 70% confluence at the time of transfection, the DNA-lipid complex was added to the cells using serum-free Opti-MEM medium. After 48–72 h of transfection, the efficiency of RNAi was detected by qRT-PCR.

The information of the primer sequences used was described as follows: ACC​CCT​GTT​TTC​AGC​TAC​GG (forward) and GGTC TTCACTCTGGCCCTTC (reverse); β-actin: TTG​TTA​CAG​GAA​GTC​CCT​TGC​C (forward) and ATGCTA TCACCTCCCCTGTGTG (reverse). Total RNA was extracted from HCT116 or panc-01 cells with TRIzol Reagent (Invitrogen, United States) in line with the manufacturer’s instructions. The CT values were further analyzed to evaluate the knockdown efficiency of siRNA.

### Gene effect on cell-line proliferation and drug sensitivity

CRISPR-based screening of genes which is significant for cancer cell-lines’ proliferation was analyzed using the DepMap database (https://depmap.org/). Meanwhile, we also evaluated the correlation between SLC3A2 expression and drug sensitivity using the Sanger GDSC2 database.

## Results

### The differential distribution of SLC3A2 expression in bulk and single-cell transcriptome analysis

**FIGURE 1:**
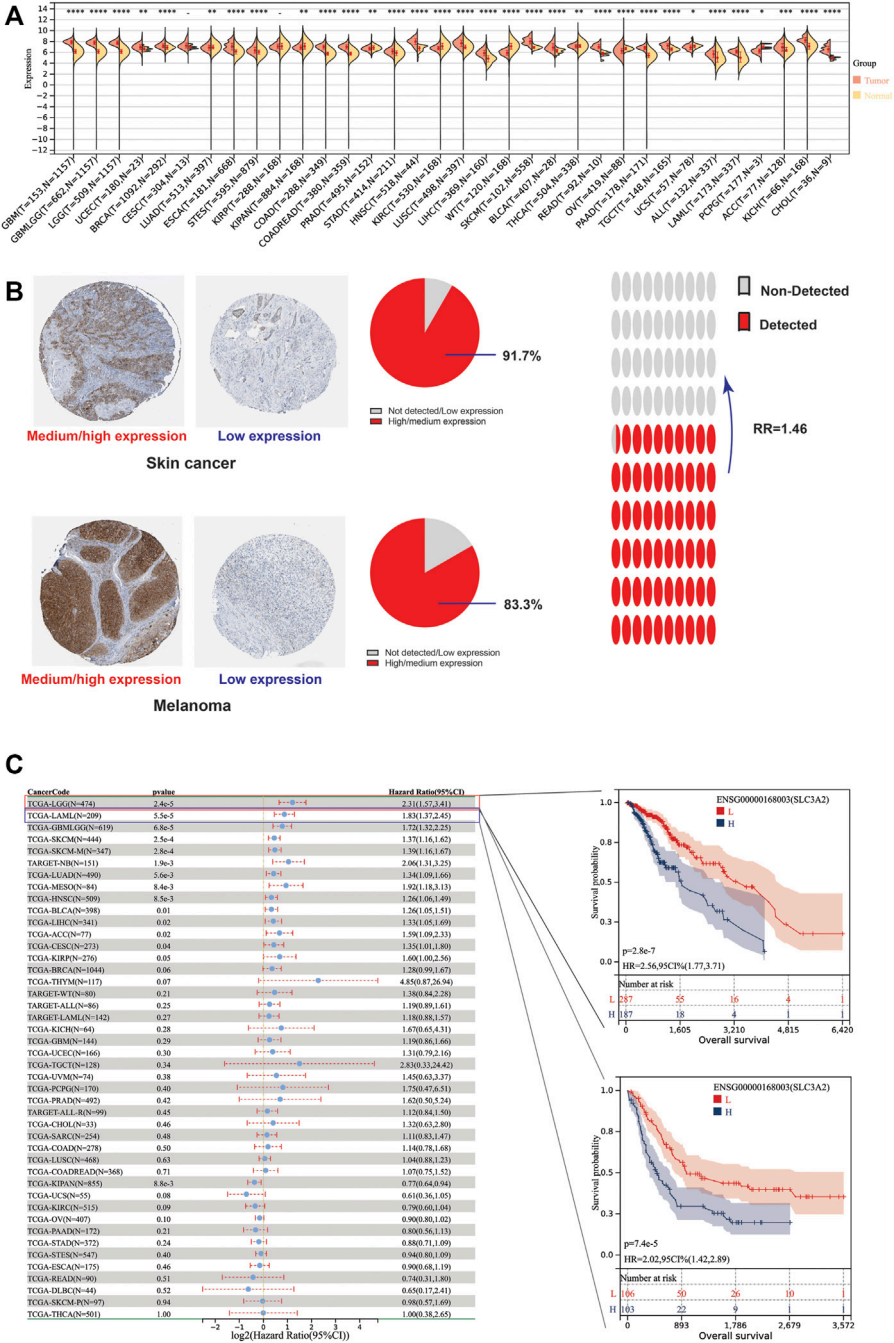
Differential expression level and prognostic implications of SLC3A2 among 34 cancers. **(A)** Differential expression of SLC3A2 between tumor and normal tissue. **(B)** Immunohistochemical staining showed higher SLC3A2 expression in cancers. **(C)** Correlation between SLC3A2 expression and OS of patients with cancer.

Due to the lack of normal samples in the TCGA cohort, we first integrated tumor samples in TCGA and normal tissues in the GTEx database, which made it more reliable to explore the differential expression of SLC3A2. The results showed SLC3A2 is upregulated in 67.6% (23/34) cancers, such as LGG, GBM, and UCEC (Figure 1A). In addition, we analyzed the protein level of SLC3A2 in tumor slices using immunohistochemical staining. For some cancers, SLC3A2 showed increased expression in cancer regions compared with normal tissues. Notably, 91.7% of SKCM showed upregulated SLC3A2 expression and 16.7% melanoma did not express SLC3A2 or showed a low expression level (Figure 1B). Overall, the ratio between high/medium expression and low expression/not detected was 1.46, which revealed that SLC3A2 may play a pivotal role in tumorigenesis.

Pan-cancer single cell analysis revealed that SLC3A2 was most abundant in malignant cells (Supplementary Figure S1A), for example in HNSC and SKCM . However, SLC3A2 was also enriched in immune cells as in glioma, which suggested that SLC3A2 may be involved in some molecular function in immune cells. In detail, SLC3A2 showed a high abundance in mononuclear macrophages and AC-like malignant cells (Supplementary Figure S1B). However, SLC3A2 was also expressed highly in fibroblasts and T cells in BRCA and NSCLC, respectively (Supplementary Figures S1C,D).

### The association between intra-tumoral SLC3A2 expression level and patient prognosis

**FIGURE 2:**
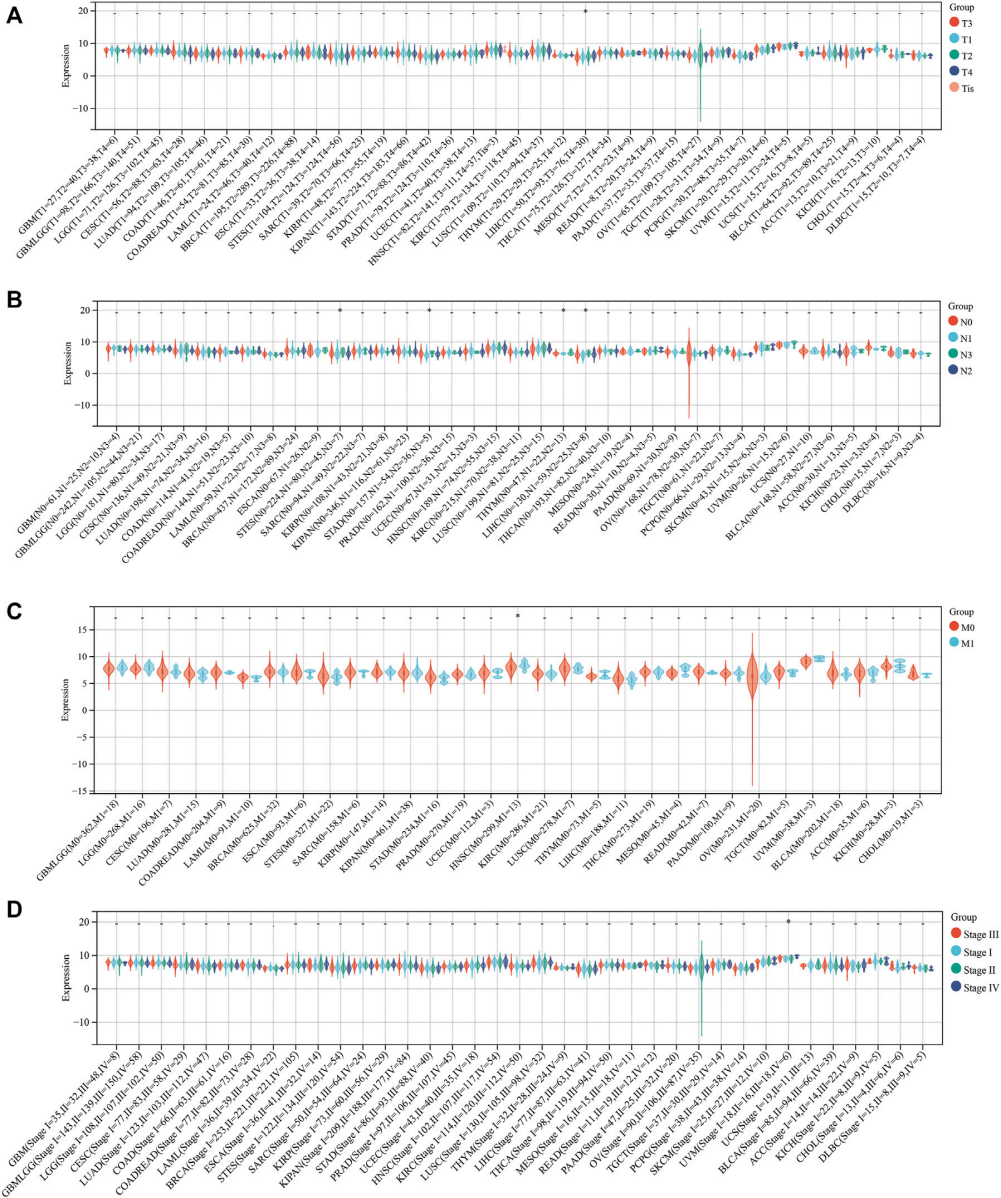
Relationship between the expression of SLC3A2 and patients’ clinical parameters. **(A)** T stage. **(B)** N stage. **(C)** M stage. **(D)** Stage.

To explore whether SLC3A2 exerted an effect on the survival interval of patients with cancer, we correlated SLC3A2 expression with patients’ OS, DSS, and DFI. Samples with a follow-up time less than 30 days were excluded from the analysis. Among the cancer types analyzed, SLC3A2 was associated with prognosis in 15 cancers, where in 93.3% (14/15) of the cases, SLC3A2 over expression was correlated with poor OS (Figure 1C). Similarly, SLC3A2 correlated with shorter DSS in 9 cancers, while only in KIRC it was associated with prolonged DSS. Notably, SLC3A2 was simultaneously related to poor OS, DSS, and DFI in ACC (Supplementary Figures S2A,B). In addition, SLC3A2 expressed the highest level in the 4^th^ grade of HNSC, which means SLC3A2 may play an important role in terminal HNSC (Supplementary Figures S3A). Likewise, for patients with LIHC, SLC3A2 had the highest expression level in the T4 stage (Figure 2A). When it comes to N stage, we found that SLC3A2 had the highest expression in N3 stage in LIHC, STAD, STES, and THYM (Figure 2B). In metastatic HNSC, SLC3A2 had higher expression compared with those that did not disseminate (Figure 2C). For stage IV UVM, SLC3A2 expressed the highest level relatively to other stages (Figure 2D). SLC3A2 also increased in the elderly patients with KIRP, KIRC, and KIPAN (Supplementary Figures S3B). These results suggest that SLC3A2 expression may be related to patients’ post-operative survival. Aberrant methylation modification either in DNA or RNA contributes to the malignant behavior of cancer cells. We also found that SLC3A2 expression was widely associated with essential regulators for various methylation modifications (m6A, m5C, and m1A) (Supplementary Figures S4A). SLC3A2 was negatively associated with the expression level of an eraser (FTO), but significantly correlated with higher expression of METTL3 and some m6A readers, which suggests SLC3A2 may be involved with enhanced m6A modification in KIPAN (Supplementary Figures S4B–E).

Given both malignant phenotype of cancer cell and tumor microenvironment could affect the clinical outcomes, we further investigated the potential role of SLC3A2 in these aspects.

### SLC3A2 is indispensable for the proliferation in multiple cancers

**FIGURE 3:**
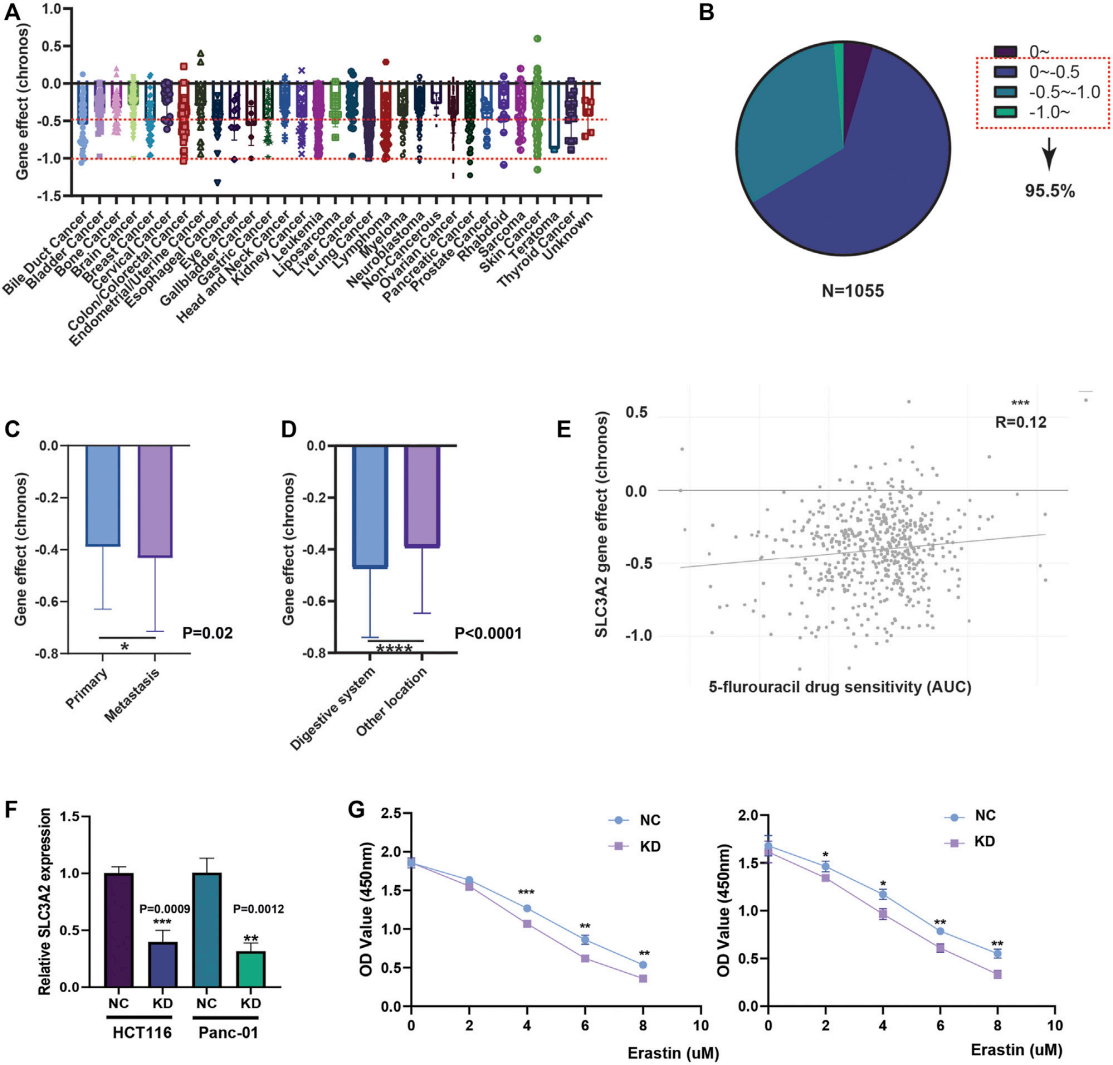
Effect of SLC3A2 on cell-proliferation and drug sensitivity in tumor cell-lines. **(A)** Gene effect of SLC3A2 in all cell-lines. **(B)** Stratification and proportion of cell-lines with different gene effect. **(C)** Metastatic cell-lines depends more on SLC3A2 expression to sustain rapid proliferation. **(D)** Digestive cell-lines depends more on SLC3A2 expression to sustain rapid proliferation. **(E)** SLC3A2 gene effect was mildly correlated with 5-FU sensitivity. **(F)** RNAi efficiency of SLC3A2 knockdown. **(G)** SLC3A2 knockdown improved sensitivity to ferroptosis in both HCT116 and Panc-01 cell-lines.

Given that SLC3A2 is associated with patients’ prognoses in multiple cancer types, we questioned whether SLC3A2 directly affects cell proliferation ability. We analyzed the CRISPR-based experimental data using DepMap and found that SLC3A2 could affect cell proliferation in the majority of tumor cell-lines (Figures 3A,B). Approximately 95% cell-lines decreased proliferative phenotypes after SLC3A2 was knocked out. Further analysis showed cell-lines derived from metastasis loci were more dependent on SLC3A2 (Figure 3C). Meanwhile, compared with other locations, cell-lines derived from the digestive system manifested more dependence on SLC3A2 expression (Figure 3D). 5-flurouracil was a classic chemotherapy drug that was widely applied in post-operative adjuvant treatment. Interestingly, the SLC3A2 gene effect was mildly correlated with 5-flurouracil resistance, which indicated that the dependence on SLC3A2 was increased in 5-flurouracil-resistant tumors (Figure 3E). We performed *in vitro* experiments to validate that SLC3A2 knockdown could improve the sensitivity to ferroptosis in a colorectal cancer cell-line and a pancreatic cancer cell-line (Figures 3F,G).

### The association between SLC3A2 expression level and tumor microenvironment remodeling

**FIGURE 4:**
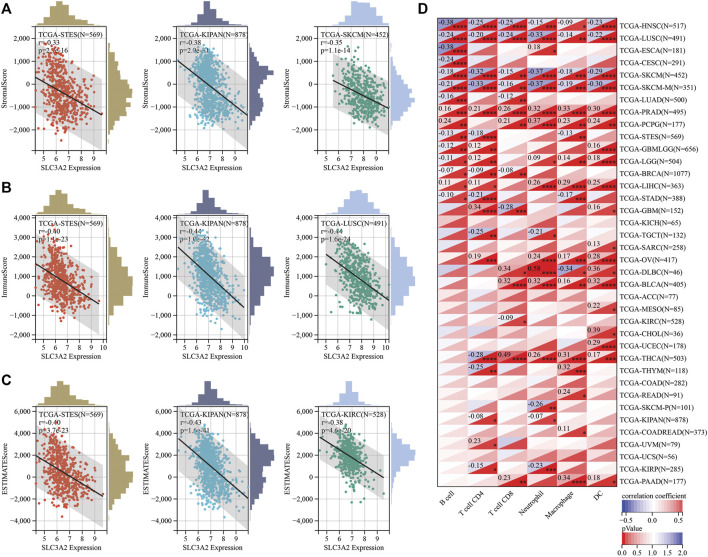
Correlation between SLC3A2 expression and constitution of microenvironment **(A–C)** SLC3A2 expression was negatively correlated with stromal score, immune score, and estimate score, respectively. Three cancer types with the largest correlation coefficient were visualized. **(D)** Correlation between SLC3A2 expression and six immune cells infiltration in cancers.

Recently, ferroptosis has been recently reported as a potential immunogenic cell death mechanism. In this context, SLC3A2 expression level may be associated with the remodeling of microenvironment in cancers. We analyzed the correlation between SLC3A2 and stromal, immune, and estimation scores for every cancer. Notably, SLC3A2 was negatively associated with these microenvironment parameters in multiple cancers, which suggested that SLC3A2 might be a culprit for the formation of “cold” tumors (Figures 4A–C). Furthermore, we evaluated the association between SLC3A2 and immune cell infiltration using different algorithms. In HNSC, LUSC, and SKCM, SLC3A2 was negatively associated with all kinds of infiltrated immune cells. On the contrary, SLC3A2 was positively correlated with immune infiltrates in PRAD and PCPG, which suggested that SLC3A2 may play distinct roles in different cancer types in terms of immune cell recruitment (Figure 4D). Notably, SLC3A2 may have different correlation among distinct immune cells. For example, SLC3A2 was positively correlated with more infiltration of CD4+T cells and dendritic cells. However, it was negatively associated with CD8^+^ T cell infiltration in GBM (Supplementary Figures S5A–C). It was validated using both the Xcell and quantiseq algorithms to show the negative correlation between CD8^+^ T cells and SLC3A2 expression (Supplementary Figures S5D). Moreover, we also found that SLC3A2 was negatively associated with the M1 signature, which was also an essential player in anti-tumor immunity (Supplementary Figures S5D). In this case, despite sufficient co-stimulatory factors and antigen-presentation in the microenvironment, anti-tumor immunity failed because of decrease in CD8^+^ T cells as the weapon to kill cancer cells. Cancer associated fibroblast (CAF) was also regarded as an important factor mediating the immune evasion in tumors. Interestingly, SLC3A2 was stringently associated with infiltrated CAFs in DLBC (*r* = 0.41, *p* < 0.01), suggesting that SLC3A2 could be a factor for fibrosis in DLBC microenvironment (Supplementary Figures S5A,B). In addition, SLC3A2 predicted decreased CD8^+^ T cells and cytotoxic lymphocytes. However, it was related to more abundant neutrophils in KIRC (Supplementary Figures S5B), which implied an immunosuppressive role of SLC3A2 in these patients.

**FIGURE 5:**
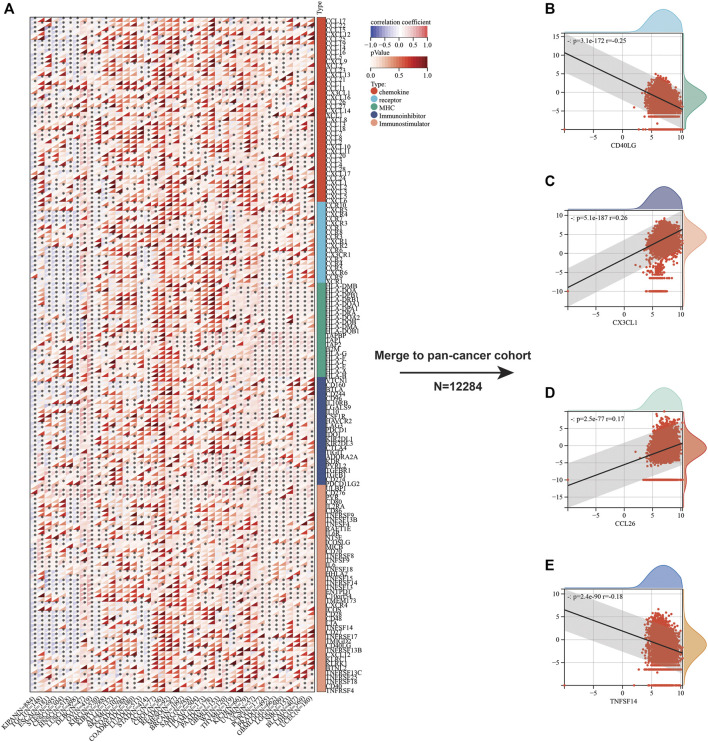
Correlation between immunoregulators and SLC3A2 expression in cancers. **(A)** Heatmap showed the correlation between each immunoregulator and SLC3A2 in every cancer. **(B–E)** Association between SLC3A2 and CD40LG, CX3CL1, CCL26, and TNFSF14 in a large pan-cancer cohort that merged by all cancer transcriptomes.

Beyond immune cell infiltrates, we also focused on the relation between immunoregulatory molecules and SLC7A11 expression. Initially, we analyzed the relationship between 150 immunoregulatory factors and SLC3A2 in each cancer type (Figure 5A). These factors comprised of five categories, including 41 chemokines, 18 receptors, 21 MHC, 24 immunoinhibitors, and 36 immunostimulators. In most cancers, as classical immunostimulators, CD40LG and TNFSF14 were negatively associated with SLC3A2. On the contrary, SLC3A2 was positively correlated with CX3CL1, which was a metastasis-related chemokine ligand. Similarly, CCL26 also showed a moderate correlation with higher SLC3A2 expression across cancer types, which has been reported to be a key element in tumor invasion and chemoresistance. These results were validated in a merged pan-cancer cohort (Figures 5B–E). Then, we analyzed the correlation between SLC3A2 expression and 60 immune check-point genes (Supplementary Figure S6A), of which 24 were stimulatory, like ICOS and CD28, while the other 36 genes were inhibitory for anti-tumor immunity, such as PD1 and PDL1 (CD274). The results demonstrated that SLC3A2 was significantly associated with PDL1 overexpression in most cancers, even in the merged cohort (*r* = 0.21, *p* < 0.0001). Similarly, SLC3A2 expression was correlated with elevated VEGFB and CD276, which were notorious targets mediating immune evasion. By contrast, in the merged pan-cancer cohort, SLC3A2 expression was significantly correlated with lower levels of GZMA and INFG, which were cytotoxic molecules that matter in anti-tumor responses (Supplementary Figures S6B–F). These findings pointed SLC3A2 as an immunosuppressive factor from a pan-cancer vision.

### The role of SLC3A2 in T-cell dysfunction and potential responses to immunotherapy

**FIGURE 6:**
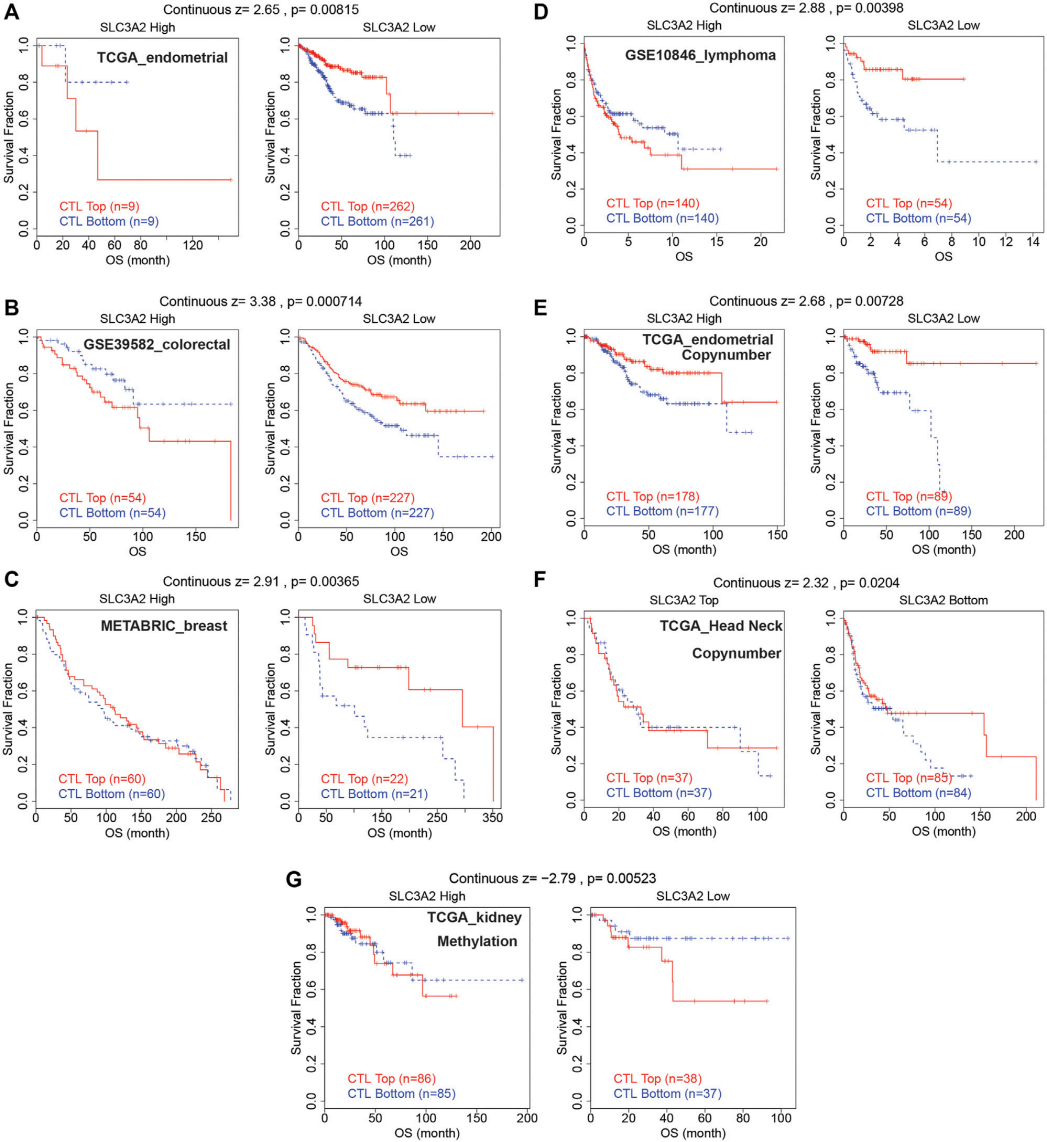
Cytotoxic lymphocyte was associated with better prognosis only in samples with low SLC3A2 expression.

The abovementioned findings suggested that SLC3A2 might be associated with alterations in the immune microenvironment. Then, we further explored whether SLC3A2 was associated with T cell dysfunction and immunotherapeutic efficacy. Using the TIDE algorithm, we found that SLC3A2 overexpression was associated with obvious T cell dysfunction. In detail, in independent cohorts for endometrial cancer, lymphoma, colorectal cancer, and breast cancer, cytotoxic T lymphocyte was associated with better overall survival only in samples with lower SLC3A2 expression (Figures 6A–D). Similarly, the copy number amplification of SLC3A2 also predicted T cell dysfunction in two independent cohorts (Figures 6E,F). By contrast, excessive methylation of SLC3A2, which reflected suppressed transcription, was associated with poor T cell function in a kidney cancer cohort (Figure 6G).

### The association between SLC3A2 and genetic parameters

**FIGURE 7:**
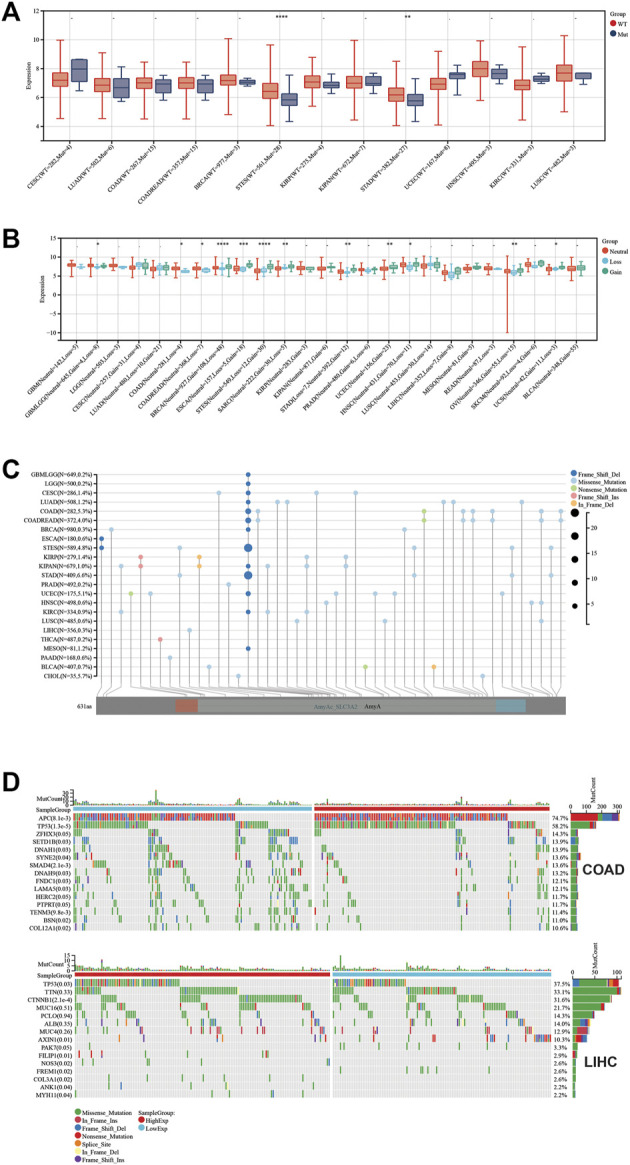
Genetic landscape regarded with SLC3A2 expression. **(A)** Association between SLC3A2 mutation and expression. **(B)** Association between SLC3A2 copy-number variation and expression. **(C)** Mutation profile of SLC3A2 across cancers. **(D)** Mutation profiles were distinct between samples with high and low SLC3A2 in COAD and LIHC.

To explore the relevance between SLC3A2 and the genetic landscape in each cancer, we first investigated whether the SLC3A2 mutation itself could affect its mRNA expression. Intriguingly, only in STES and STAD, SLC3A2 mutations corresponded to reduced transcriptomic levels, which was possibly attributed to the low mutated rate in other types of cancer (Figure 7A). The copy number variation well reflected the transcriptomic level of SLC3A2 in most cancer types. In brief, higher expression of SLC3A2 was expected to appear in samples with gain variation compared to loss variation or wild type. It indicated that SLC3A2 was not influenced dramatically by post-transcriptomic regulation (Figure 7B). The total mutation rate of SLC3A2 ranged from 0.7 to 6.6%, in which missense variation was the most common variation of SLC3A2 across cancers (Figures 7C,D).

**FIGURE 8:**
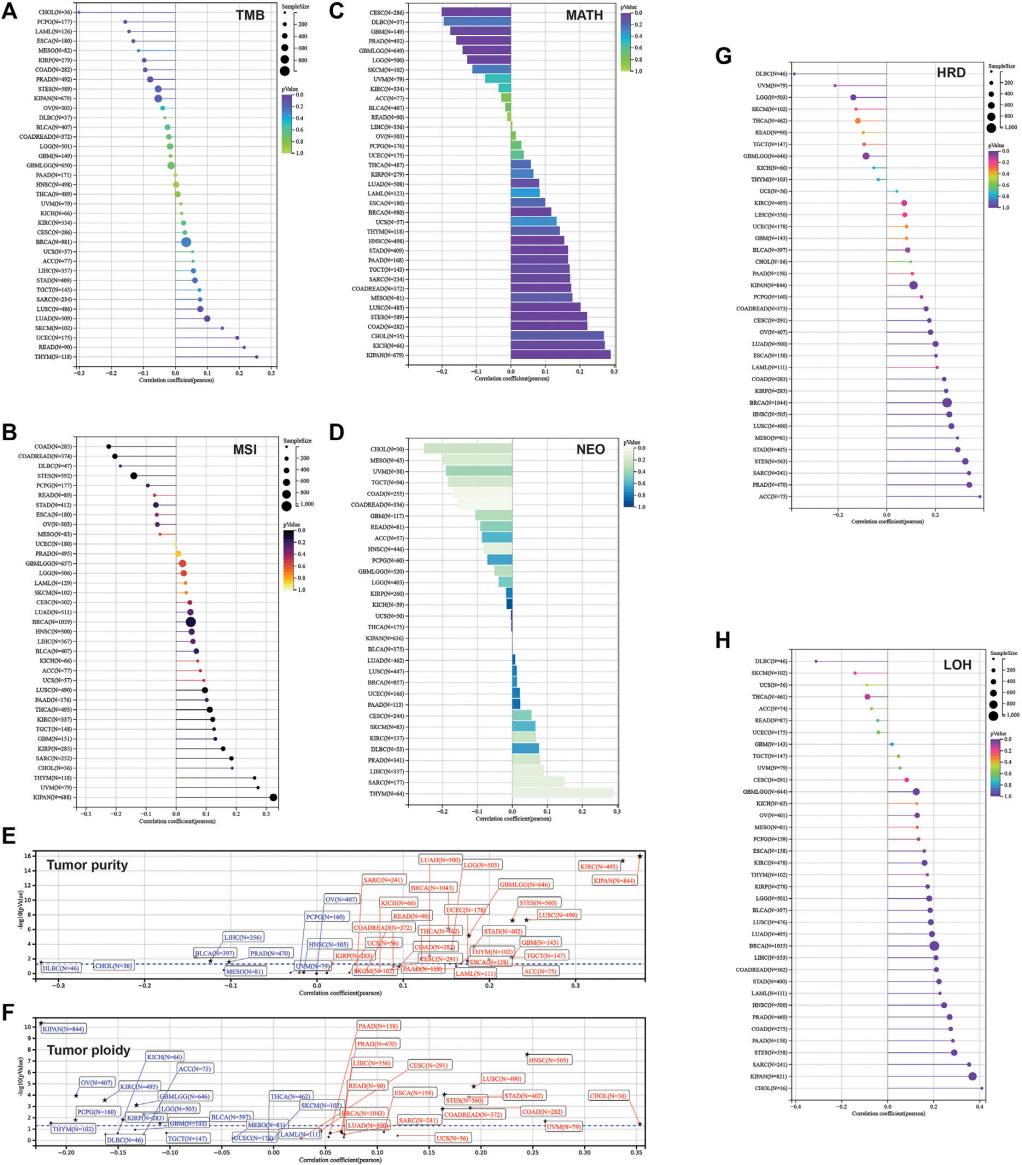
Correlation between parameters of genetic instability and SLC3A2 expression. **(A)** TMB. **(B)** MSI. **(C)** MATH. **(D)** NEOANTIGEN. **(E)** Tumor purity. **(F)** Tumor ploidy. **(G)** HRD. **(H)** LOH.

Furthermore, we calculated the mutant-allele tumor heterogeneity (MATH), landscape of microsatellite instability (MSI), tumor mutation burden (TMB), neoantigen, purity, ploidy, homologous recombination deficiency (HRD), and loss of heterozygosity (LOH) for each sample across cancers. Then, correlation analysis was performed in terms of these parameters and SLC3A2 expression (Figures 8A–H). Notably, SLC3A2 was significantly associated with higher TMB, MSI, and neoantigen in THYM, which meant more events of immunosurveillance would appear in this type of cancer. In addition, SLC3A2 was positively associated with HRD in multiple cancers, especially in ACC. In this context, SLC3A2 targeting could be considered when applying PARP inhibitors. Overall, the landscape of gene mutation in samples with high and low SLC3A2 expression was different, which may be explained by distinct drive genes for each cancer. However, some genes had a higher mutation rate in SLC3A2-high samples. For example, TP53 mutation appeared more in samples with higher SLC3A2 expression in COAD and LIHC.

## Discussion

The xCT complex is a major target for ferroptosis inducers and is elevated in cancer cells. Ferroptosis inducer “erastin” targets the xCT complex to trigger ferroptosis *in vitro* and *in vivo* (Zhao et al., 2020; Koppula et al., 2021; Liu et al., 2022; Wiernicki et al., 2022). This complex is comprised of two units, which are SLC7A11 and SLC3A2 (Koppula et al., 2021). Among them, numerous studies have uncovered the role of SLC7A11 in cancer development. However, less attention has been paid to the latter.

Only few studies initially revealed that SLC3A2 contributes to initiation and development in carcinogenesis. It is necessary to explore the function of SLC3A2 in patients’ tumor samples. Therefore, it is imperative to explore the expression and clinical relevance of SLC3A2 in different cancer types. In this study, we found that SLC3A2 predicted worse prognosis in multiple cancers, which was consistent with previous studies. For example, we revealed a worse prognosis associated with SLC3A2 in LUAD and LIHC, which was consistent with some previous reports (Shin et al., 2018; Li et al., 2019; Ma et al., 2021). While Zhu et al. revealed that SLC3A2 was upregulated in human osteosarcoma and promoted disease progression *via* the PI3K/Akt signaling pathway (Zhu et al., 2017), our study did not find SLC3A2 could reflect a better survival interval for patients with SARC. To validate the effect of SLC3A2 on cell proliferation, we analyzed the results from a large-scaled CRISPR-based screening in DepMap and found that the growth of around 95% cell-lines was partially dependent on SLC3A2 (Liu et al., 2022; Wiernicki et al., 2022), which supported the essential role of SLC3A2 in fueling cancer cell proliferation. Indeed, SLC3A2 was involved in the transportation of cysteine and glutamate, which maintained the resistance to ferroptosis. It was assumed that tumor cells faced a lot of pressure from ferroptosis induction *in vivo*, such as chemotherapy (Zhang et al., 2020), radiotherapy, and immune cell killing (Lang et al., 2019). In this context, it is plausible for SLC3A2 to maintain the proliferative phenotype of cancer cells *in vivo*. However, it could not explain why SLC3A2 could promote the proliferation of cell-lines *in vitro*, which implied another mechanism by which SLC3A2 activated the proliferative ability beyond ferroptosis pathways.

Immunotherapy has recently emerged as a very effective new therapy and has become a powerful clinical strategy for treating cancer (Lu and Robbins, 2016; Marabelle et al., 2017; Riley et al., 2019). The number of immunotherapy drug approvals has been increasing, with numerous treatments in clinical and preclinical development (Ngwa et al., 2018; Liu et al., 2019). Despite this success, immunotherapy only works in a subset of cancers, and only a fraction of patients with cancer respond to immunotherapy. Therefore, it is important to consider the role of SLC3A2 in immunotherapy in distinct cancer types. Few studies have researched the role of SLC3A2 in anti-tumor immunity. Ikeda et al. reported that SLC3A2 was essential for the functioning of regulator T cells, although it did not yield from cancer research, but it could be estimated that SLC3A2 expressed on Tregs could facilitate its immunosuppressive function and promote immune evasion (Ikeda et al., 2017). In addition, SLC3A2 could also serve as a target for CAR-T therapy given its overexpression on the membrane of cancer cells (Pellizzari et al., 2021).

Here, we conducted a bioinformatic study at pan-cancer level to systematically explore whether the intra-tumoral expression of SLC3A2 is associated with patients’ survival and its potential value in immunotherapy. This study has several strengths to declare. First, SLC3A2 is a promising anti-cancer target in the future. Our study was the first to comprehensively analyze the role of SLC3A2 at pan-cancer level, which provided many valuable and integrated advice for the selection of appropriate cancer types. Second, analysis based on human samples made our results more credible compared with previous studies that explored the SLC3A2 function at cell or animal levels. Third, we explored the association between SLC3A2 expression and potential response to immunotherapy at a pan-cancer level, which has an important reference value in judging patients’ condition, estimating prognosis, directing treatment, and evaluating the curative effect. Certainly, this study has some limitations. On one hand, the present study lacks pathological samples for validation. However, as a pan-cancer study, it is difficult to validate the findings using tumor tissues of all origins. The dataset we used in the present study was well-designed and widely tested, which supported the reliability of our study. On the other hand, whether SLC3A2 expressed in tumors affects the tumor microenvironment was clarified in the present study. Future studies are encouraged to explore the underlying mechanism by which SLC3A2 affects anti-tumor immunity.

In conclusion, this pan-cancer level bioinformatic study systematically elucidated the role of intra-tumoral expression of SLC3A2 in the survival of cancer patients and potential immunotherapeutic response. Our results helped in identifying a new biomarker for detection and monitoring of cancer. Future research into the biological mechanism could further help with targeted treatment for cancer patients.

## Data Availability

The original contributions presented in the study are included in the article/Supplementary Material; further inquiries can be directed to the corresponding authors.
