# Supplementary figures and images for: Characterizing the role of SLC3A2 in the molecular landscape and immune microenvironment across human tumors
Source: Front Mol Biosci. 2022 Aug 5;9:961410. doi: 10.3389/fmolb.2022.961410 (PMC9388758; doi:10.3389/fmolb.2022.961410)

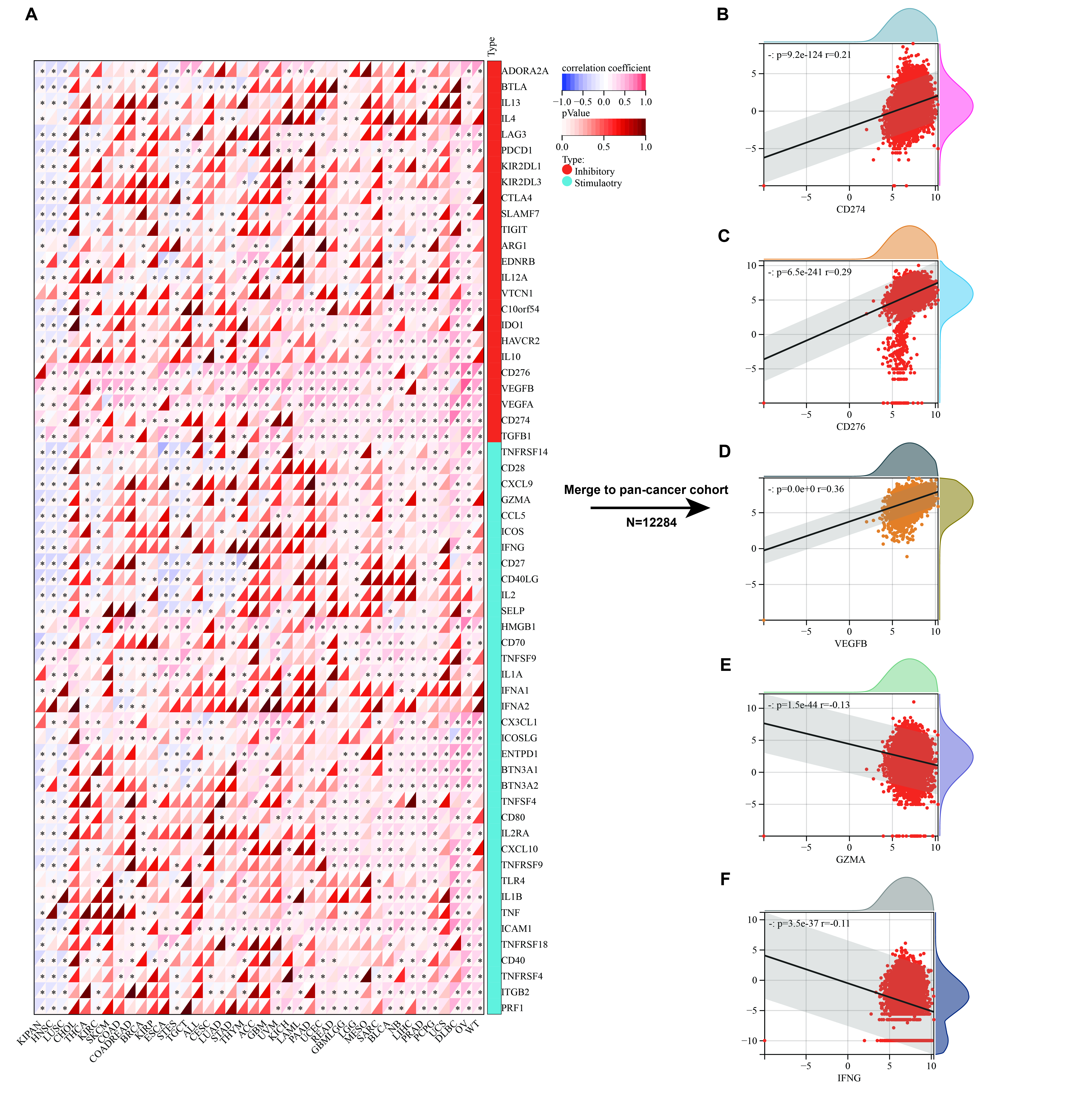

Supplement: Supplementary file 1 [file Figure14.JPEG]

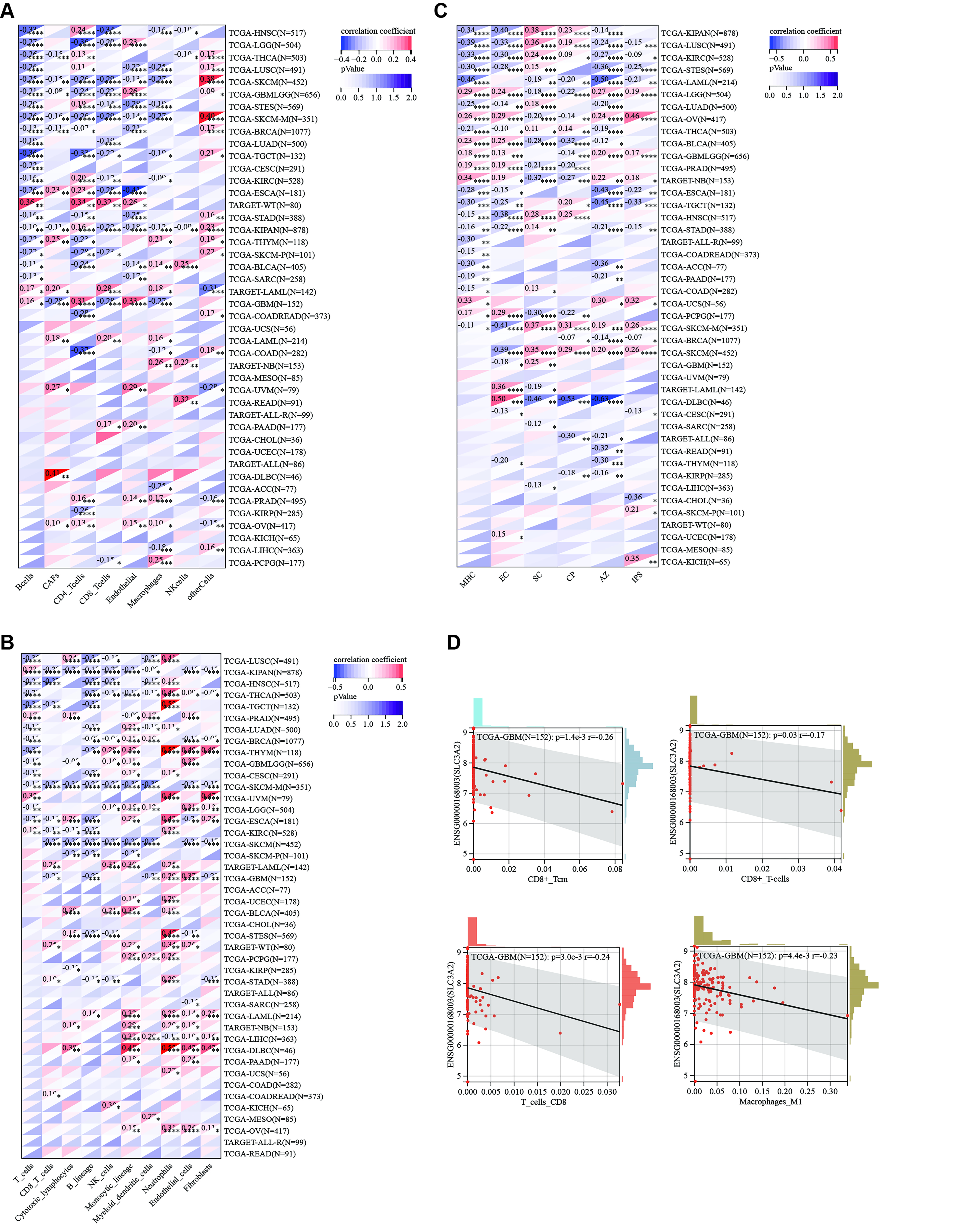

Supplement: Supplementary file 3 [file Figure13.JPEG]

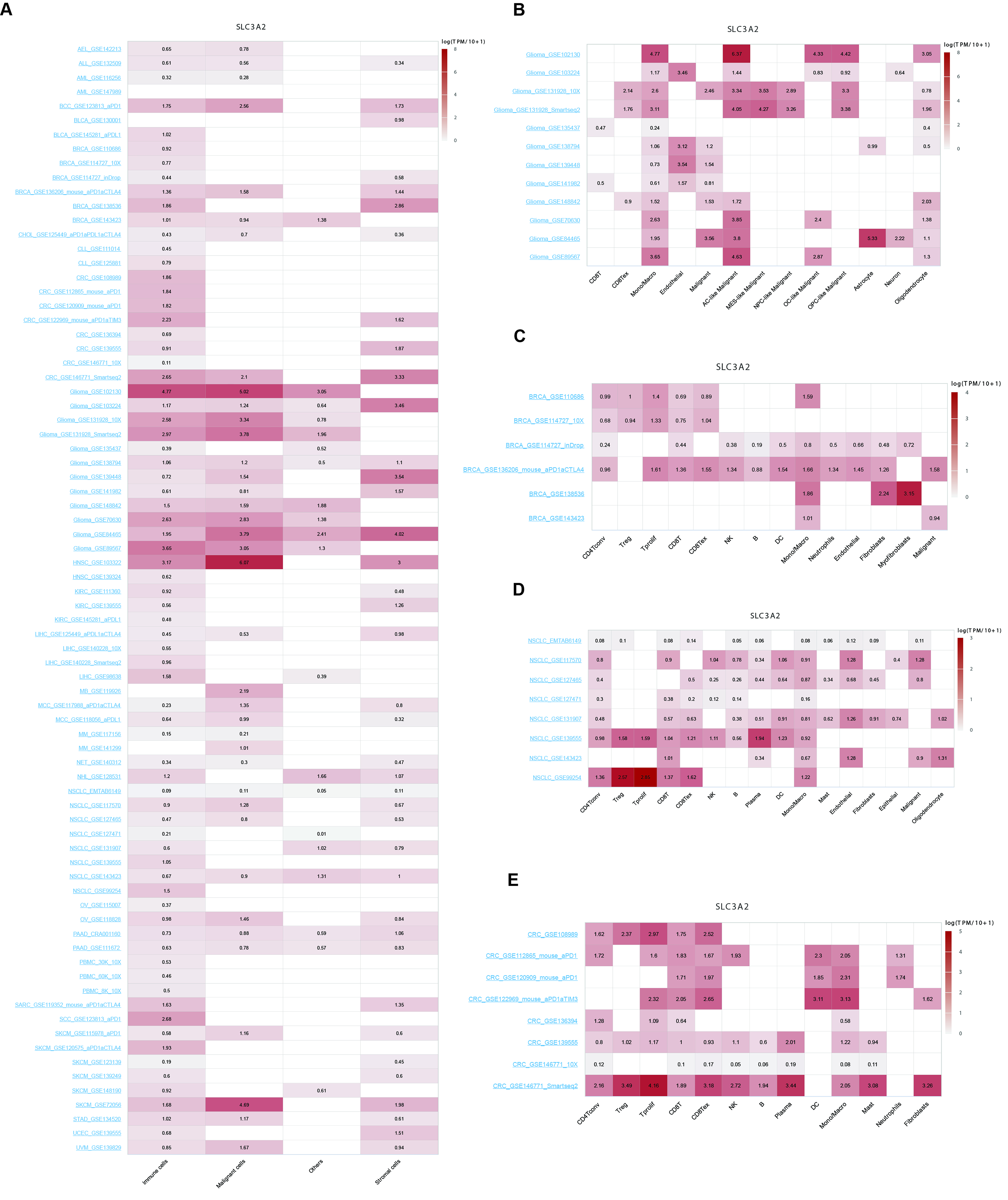

Supplement: Supplementary file 4 [file Figure9.JPEG]
